# System Performance Check (SPC): A Readiness Companion to Multi-Attribute Method (MAM) Analysis in Biopharmaceutical Quality Control

**DOI:** 10.3390/ph19081240

**Published:** 2026-08-06

**Authors:** Jahziel Chase, Melissa Sato, Gordon Slysz, Mahsan Miladi, Mike Knierman, Robert Barkovich, Jared Auclair

**Affiliations:** 1 Center of Bio-Innovation, Northeastern University, 147 South Bedford Street, Burlington, MA 01803, USA; chase.j@northeastern.edu; 2 Department of Chemistry and Chemical Biology, Northeastern University, 360 Huntington Avenue, Boston, MA 02115, USA; 3 Barnett Institute of Chemical and Biological Analysis, Northeastern University, 360 Huntington Avenue, Boston, MA 02115, USA; 4 Agilent Technologies, Inc., Santa Clara, CA 95051, USA; gordon.slysz@agilent.com (G.S.); mahsan.miladi@agilent.com (M.M.); mike.knierman@gmail.com (M.K.); robert.barkovich@agilent.com (R.B.)

**Keywords:** multi-attribute method, system performance check, critical quality attributes, peptide mapping, LC-MS, system suitability, USP 1060, biopharmaceutical quality control, mass spectrometry

## Abstract

**Background/Objectives:** The multi-attribute method (MAM) is a powerful LC-MS workflow for site-specific monitoring of critical quality attributes (CQAs) in biopharmaceutical products, but its sensitivity to LC drift, ionization variability, and mass-calibration shifts has slowed adoption in routine quality control. A blank injection confirms that the system is clean; it does not confirm that the system is ready. This work evaluates a system performance check (SPC) workflow, built around a 13-peptide LC/MS reference standard designed against the system-readiness metrics in USP General Chapter 1060, as a readiness assessment performed before MAM sample analysis. **Methods:** The workflow was run on a recently installed Agilent 6230C LC/TOF with OpenLab CDS and the integrated MAM for OpenLab CDS, following routine instrument tune and calibration. The 13-peptide mix probes mass accuracy, retention time, peak area and height, in-source fragmentation, methionine oxidation, chromatographic resolution, and sodium and iron adduct formation; it also includes a heavy-to-light peptide pair for a single-point, low-abundance response check and a deamidated peptide pair to monitor a clinically important post-translational modification. Triplicate injections were evaluated against per-target acceptance criteria: mass accuracy (±10 ppm or ±13 ppm for EYK), retention-time %RSD (≤0.5%), and, for a subset of targets, peak-area %RSD (≤10%). **Results:** Across the three replicates, all thirteen peptides met every acceptance criterion applied to them. The twelve primary targets met their mass-accuracy and retention-time criteria, and the relative-area monitors (sodium and iron adducts, methionine oxidation, the heavy/light detector check, and the deamidation pair) met theirs. The asparagine deamidation pair was detected and reproducibly measured as a relative-area ratio at the +2 and +3 charge states in every replicate (6.7 to 7.0% at +2 and 7.5 to 7.8% at +3, against a <8% criterion), demonstrating reproducible relative monitoring of a clinically important post-translational modification. **Conclusions:** The SPC workflow provides a single, evidence-backed readiness measurement prior to MAM sample analysis, and it produces documentation, including the audit trail and electronic-signature functionality in OpenLab CDS 3.0, aligned with data-integrity expectations.

## 1. Introduction

Biopharmaceutical characterization has historically relied on a stack of orthogonal assays such as ion-exchange chromatography, capillary electrophoresis, and ligand-binding methods, each reporting on a narrow slice of product quality [1]. As therapeutic modalities have grown more complex, these legacy assays increasingly miss the kind of site-specific information needed to track post-translational modifications (PTMs) and sequence variants in protein therapeutics, fusion proteins, antibody–drug conjugates, and the emerging family of cell and gene therapy products [1,2,3]. Industrial groups have responded by consolidating product quality attribute monitoring into a single LC-MS workflow known as the multi-attribute method (MAM), which combines targeted CQA quantitation with non-targeted new peak detection (NPD) in one analytical run [4,5,6,7,8].

MAM has three properties that make it attractive for routine QC. First, it consolidates assays. A single tryptic digest mapped by LC-MS gives quantitative readouts on glycosylation, oxidation, deamidation, C-terminal lysine clipping, and other CQAs defined under ICH Q8(R2) [9], replacing several compendial assays in the process [4,5,10]. Second, it measures PTMs at the residue level, with the sensitivity and specificity of high-resolution mass spectrometry, rather than reporting them indirectly through bulk physicochemical proxies [11,12]. Third, the workflow is increasingly automated end to end, from sample preparation through report, which makes it tractable for batch release rather than only for development [13,14]. Industrial case studies from Genentech, Amgen, Pfizer, and Biogen have shown that MAM can substitute for several conventional release assays while matching or improving on their quantitative performance [4,15,16,17].

These strengths, however, come with a corresponding sensitivity. MAM depends on consistent enzymatic digestion, stable chromatographic retention, well-behaved electrospray ionization, and a mass spectrometer that holds its calibration, and small deviations in any of these dimensions can manifest as misquantified PTMs or as false NPD hits [10,16,18,19]. Thus, the community has converged on the view that bringing MAM into routine QC requires a more thorough system-suitability framework than the single-compound checks that suffice for many other LC-MS workflows [4,10,20,21]. Inter-laboratory studies and industry experience point in the same direction, suggesting that the reliability of MAM tracks closely with the reliability of the platform on which it runs [20].

The system performance check (SPC) is the readiness assessment that the USP 1060 guidelines recommend precedes an MAM analysis. It uses a curated mixture of peptides that span the chromatographic and ionization range a typical tryptic digest will occupy, and it evaluates several performance dimensions in parallel, namely mass accuracy, retention time, peak area, and peak height [4,15]. The output is a single pass-or-fail verdict indicating whether the LC/MS system and the data processing pipeline are operating as expected before MAM sample analysis begins. Unlike a tuning compound or a single-peptide check, the SPC mixture interrogates the system with multiple analytes across the run simultaneously, which most likely makes it more sensitive to detect the subtle drift that appears first in regions of the chromatogram not covered by single-marker tests [20]. The same metrics that drive the per-session readiness assessment can subsequently be trended across days and instruments to flag column aging, source contamination, or calibration drift before these issues reach a product sample; however, such longitudinal trending is positioned here as a future direction rather than a claim of the present study.

**Figure 1:**
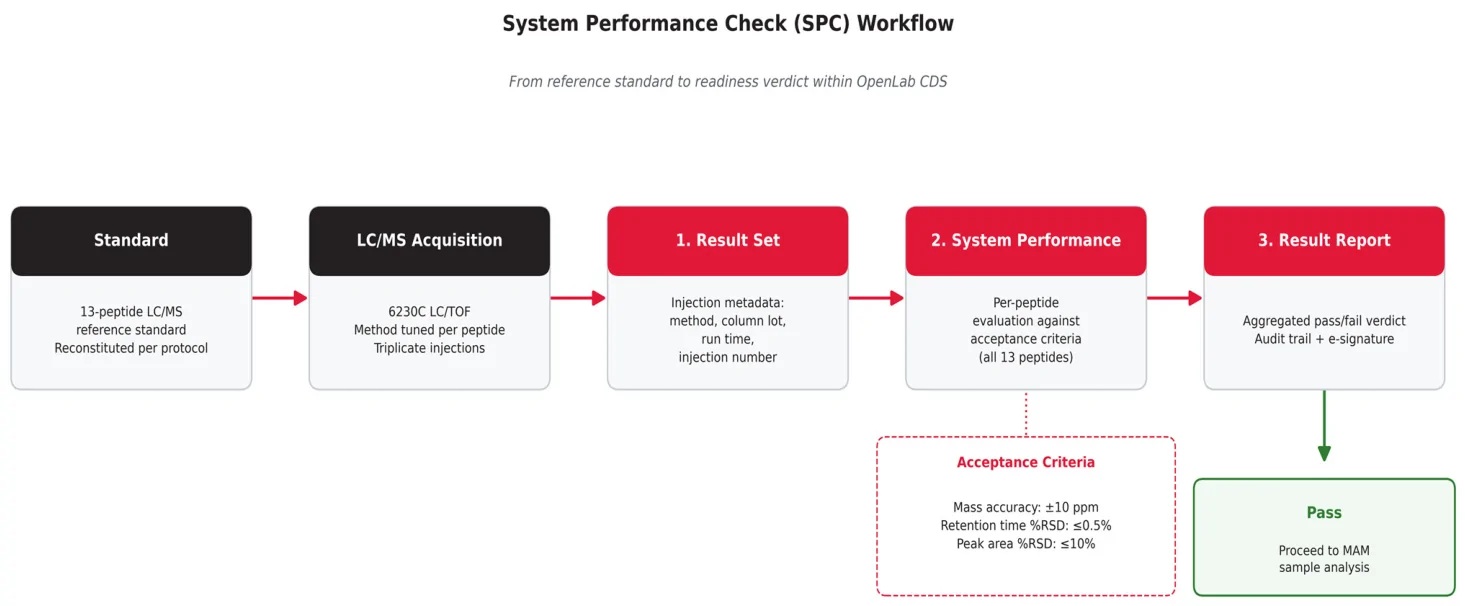
Overview of the system performance check (SPC) software workflow. The 13-peptide reference standard is reconstituted and injected in triplicate on the Agilent 6230C LC/TOF using an LC-MS acquisition method with parameters tuned for this specific peptide mixture to maximize signal intensity and stability across all 13 peptides. The resulting acquisition then passes through three stages within OpenLab CDS: the result set page (injection metadata), the system performance page (per-peptide evaluation against acceptance criteria), and the result report (aggregated pass-or-fail verdict suitable for compliance documentation).

Here we evaluate the SPC workflow on an Agilent 6230C LC/TOF MAM platform (Agilent Technologies, Santa Clara, CA, USA) with OpenLab CDS 3.0, using the 13-peptide Agilent reference standard described in Section 4.1. Our objectives were straightforward: (1) confirm that all 13 peptides could be identified and detected as well-defined chromatographic peaks in a single injection on the platform; (2) evaluate triplicate injections of the standard against pre-defined acceptance criteria for mass accuracy, retention time, and peak area; (3) confirm that the deamidation pair (VVSVLTVLHQDWLNGK and its deamidated counterpart VVSVLTVLHQDWLDGK) was detected and quantified, since reliable tracking of a deamidation event is one of the most analytically meaningful demonstrations the standard can make; and (4) characterize the workflow as the daily readiness check that supports routine MAM operations. The work is framed as the foundation on which longitudinal SPC trending and extension to therapeutic protein samples will be built. An overview of the complete SPC workflow, from standard reconstitution through the automated readiness verdict, is shown in Figure 1.

## 2. Results

### 2.1. Peptide Identification and Chromatographic Performance

**Figure 2:**
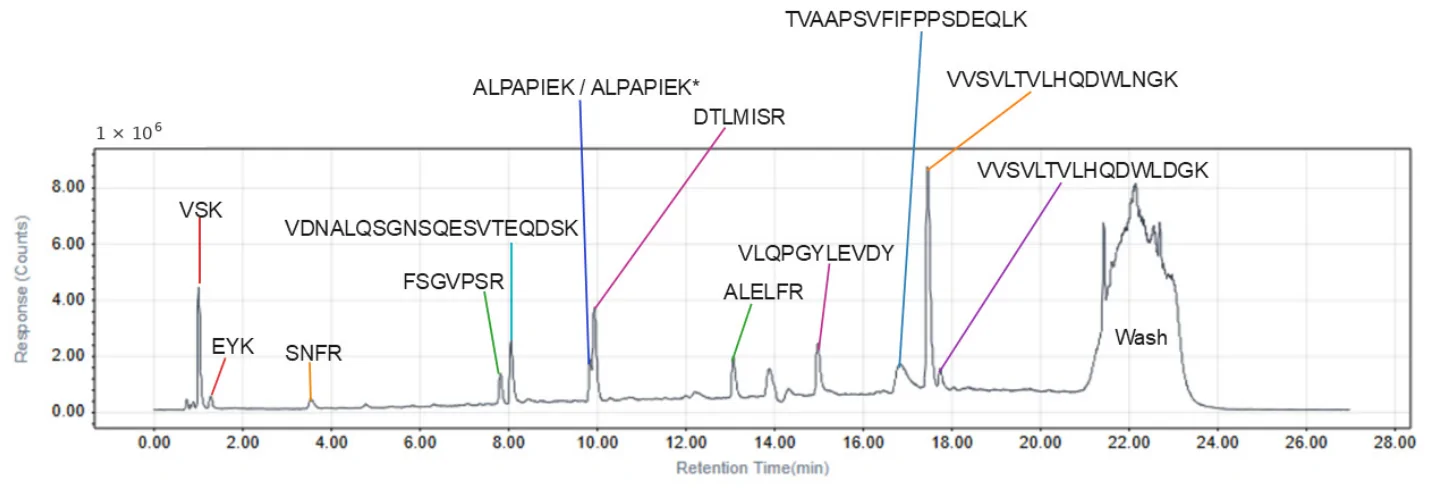
Total ion chromatogram (TIC) of a representative injection of the 13-peptide reference standard, acquired on the Agilent 6230C LC/TOF, showing the peptides eluting across the gradient. Peptides are labeled at their respective retention times, from the early-eluting hydrophilic peptides (VSK, EYK, SNFR) through the late-eluting deamidation pair (VVSVLTVLHQDWLNGK and VVSVLTVLHQDWLDGK). The broad signal eluting after approximately 21 min corresponds to the high-organic column wash and equilibration segment of the gradient and is not part of the peptide standard. The asterisk in ALPAPIEK* denotes the isotopically labeled heavy internal standard (^13^C_6_, ^15^N_2_ lysine).

Injection of the 13-peptide reference standard on the 6230C LC/TOF under the conditions in Section 4.3 produced well-defined chromatographic peaks for all 13 peptides. To provide a visual representation of the peptides eluting across the gradient, identifications were confirmed by Find by Formula in Agilent MassHunter Qualitative Analysis 12.0, with retention times consistent across replicates, mass accuracy within the instrument specification, and acceptable peak shape. This identification check was performed for visualization and confirmation only and was independent of the SPC pass-or-fail evaluation, which was carried out within the MAM for OpenLab CDS (Section 4.5). A representative total ion chromatogram showing all 13 peptides across the gradient is shown in Figure 2.

### 2.2. Detection and Reproducibility of the Deamidated Peptide Pair

**Figure 3:**
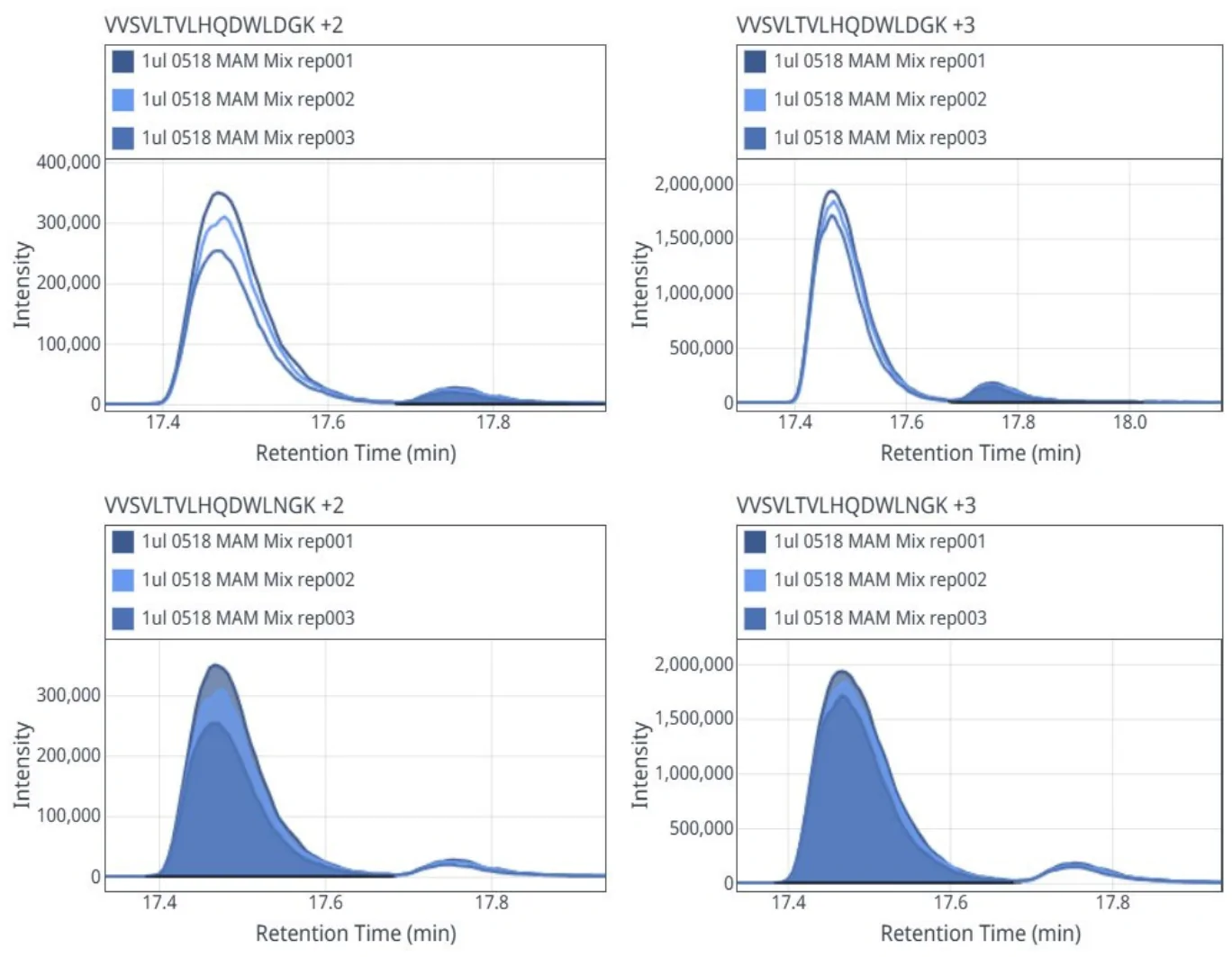
Extracted ion chromatograms of the deamidation-pair peptides within the system performance workflow, shown across the three replicate injections of the 13-peptide standard. The deamidated peptide VVSVLTVLHQDWLDGK (17.75 min) and the native peptide VVSVLTVLHQDWLNGK (17.47 min) are each monitored at the [M+2H]^2+^ and [M+3H]^3+^ charge states. The three replicate traces overlap closely, indicating reproducible retention and signal intensity for both peptides; the pair elutes as a closely spaced cluster spanning approximately 17.4 to 17.8 min.

The deamidation-pair peptides were detected and reproducibly monitored across the triplicate-injection set. As shown in Figure 3, the native VVSVLTVLHQDWLNGK (eluting at 17.47 min) and the deamidated VVSVLTVLHQDWLDGK (eluting at 17.75 min) were each extracted at the [M+2H]^2+^ and [M+3H]^3+^ charge states, with the three replicate traces overlapping closely and the pair eluting as a closely spaced cluster spanning approximately 17.4 to 17.8 min. The deamidated form was measured as a deamidation-pair relative area of 6.7 to 7.0% at the +2 charge state and 7.5 to 7.8% at the +3 charge state across the three replicates (criterion < 8%), placing the deamidation level within its acceptance range at both charge states. This is a reproducible relative measurement rather than a certified quantitative value, as no reference deamidation level is assigned to the standard. Reproducible monitoring of this region is analytically important because deamidation is among the most clinically relevant PTMs in biotherapeutic characterization [11]; thus, the consistency of retention and signal for the deamidation-pair peptides across replicates supports confidence in the downstream MAM measurement of this attribute.

### 2.3. Triplicate-Injection System-Suitability Performance

**Figure 4:**
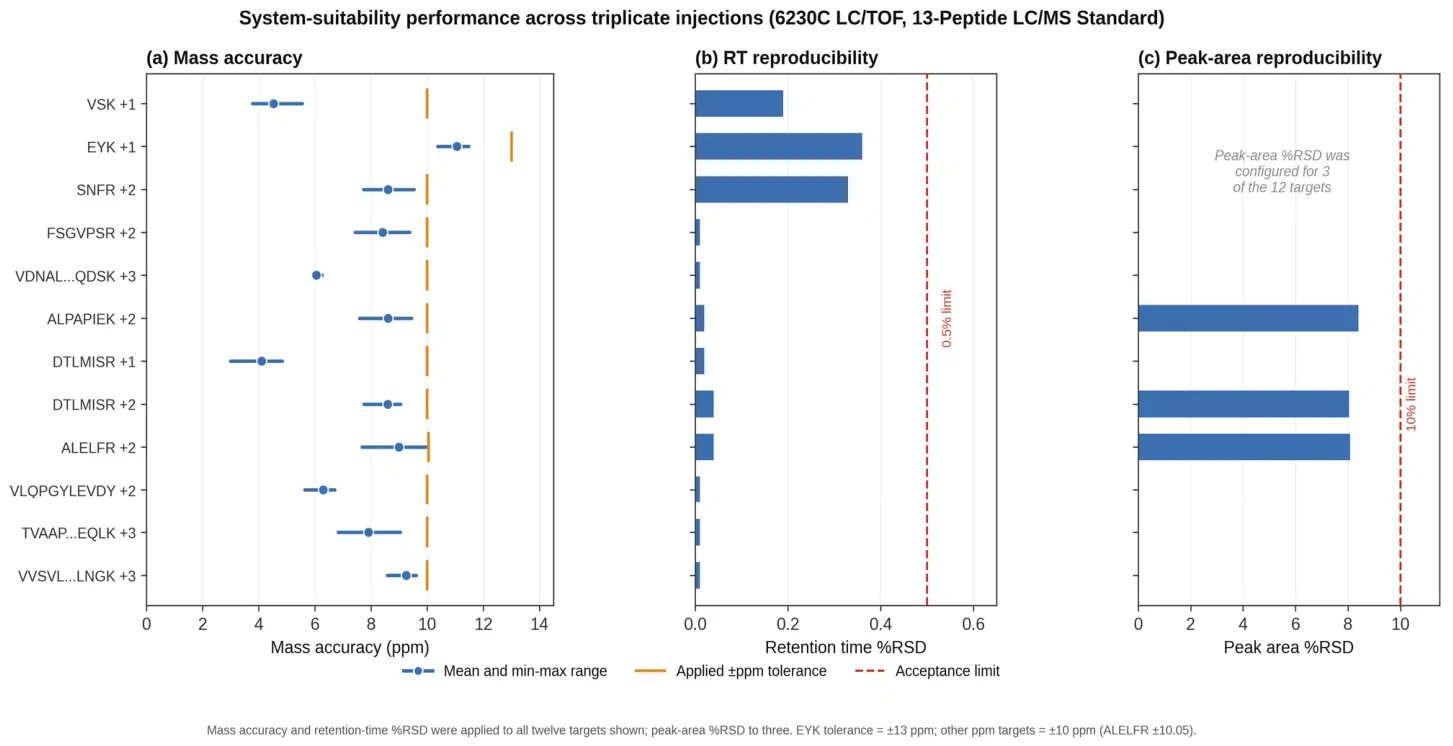
Quantitative system-suitability performance across the triplicate-injection set on the Agilent 6230C LC/TOF. (**a**) Mean mass accuracy for each target (point) with the minimum-to-maximum range (bar) and the applied per-target tolerance (tick): ±10 ppm for most targets, ±13 ppm for EYK, and ±10.05 ppm for ALELFR. (**b**) Retention-time %RSD for each target against the ≤0.5% limit. (**c**) Peak-area %RSD against the ≤10% limit for the three targets to which that criterion was applied. All applied criteria were met for every target.

The 13-peptide standard was injected in triplicate within a single analytical session, and each peptide was evaluated against the pre-defined acceptance criteria. All peptides passed across the three replicate injections. Retention-time stability was strong across the entire gradient, with %RSDs well below the 0.5% threshold. Mass accuracy was within the ±10 ppm window for the peptides evaluated on a ppm basis, while the heavy internal standard and the oxidized variant were confirmed by exact mass. Peak-area %RSDs remained below the 10% threshold for the three peptides to which that criterion was applied. Absolute peak areas showed a modest, largely monotonic decline across the three injections (rep001 to rep003) for many targets. A decline in precursor peak area can arise from several factors, including ionization efficiency, ion transmission, and in-source fragmentation, and the root cause was not specifically investigated in this single-session study; the trend is acknowledged without assigning a mechanism. This variation is captured within the reported area %RSD values, and the complete per-replicate areas for all thirteen peptides are provided in Table S1 so that the trend can be assessed directly. The per-peptide replicate overlays for the full panel are shown in Figure 4, and representative results spanning the principal diagnostic categories are summarized in Table 1.

### 2.4. Aggregated Readiness Verdict

**Figure 5:**
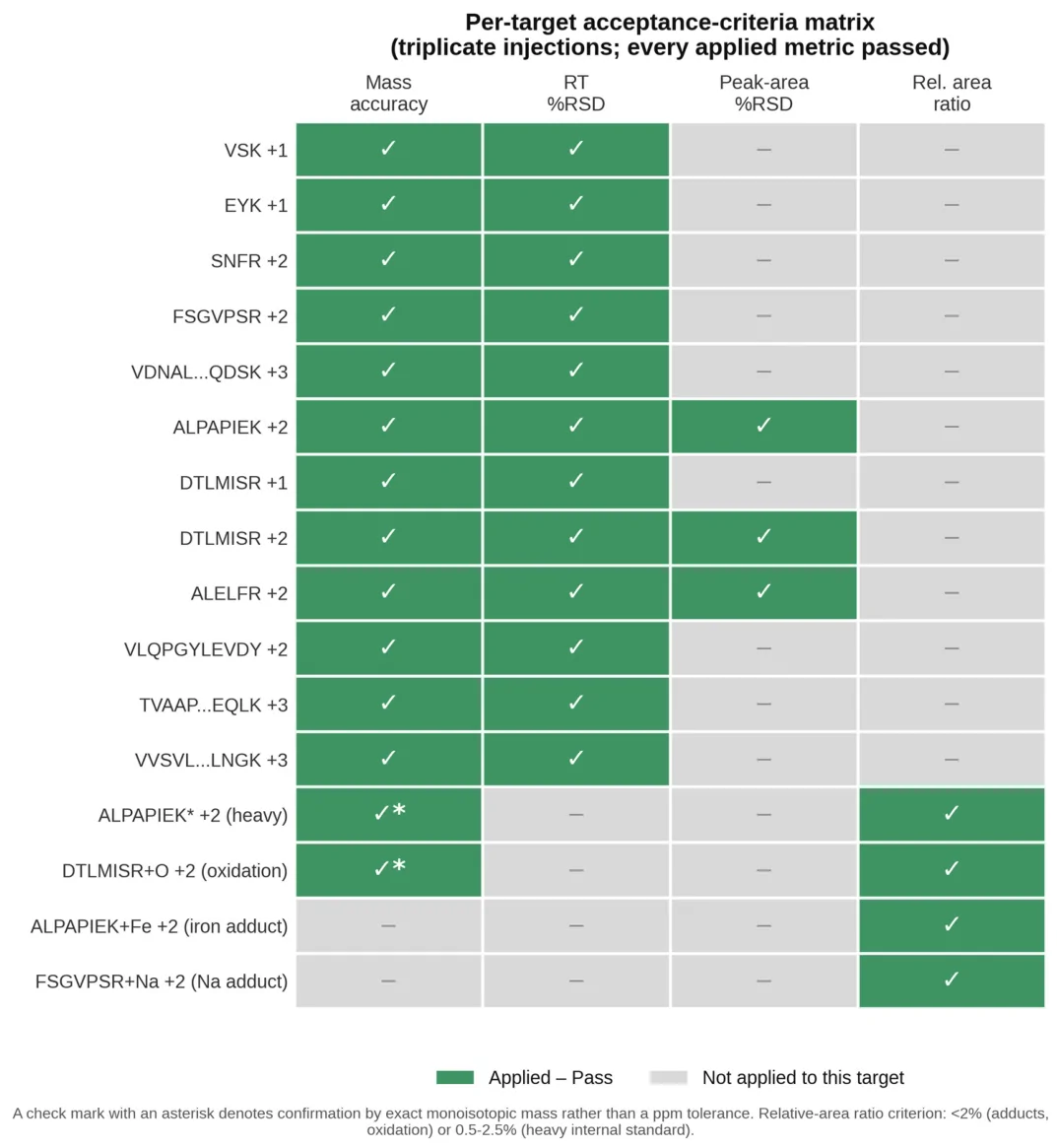
Per-target acceptance-criteria matrix for the triplicate-injection set. Each cell shows whether a given metric (mass accuracy, retention-time %RSD, peak-area %RSD, or relative-area ratio) was applied to a target and, where applied, that it passed; a dash marks a metric not applied to that target. The heavy internal standard and the oxidized variant are confirmed by exact mass, and the sodium and iron adducts by relative-area ratio. Every applied criterion was met, yielding an overall pass generated within OpenLab CDS with audit trail and electronic signature support.

Aggregating across all 13 peptides and all three metrics, the MAM for OpenLab CDS returned a pass verdict for the triplicate-injection set (Figure 5). This verdict is the operational output of the SPC workflow and the practical answer to the question that motivated the work: is the system ready to produce reliable MAM data on the samples that follow? In this evaluation the answer was yes.

## 3. Discussion

### 3.1. SPC as a Readiness Check for MAM

MAM is sensitive to upstream system performance in a way that a blank injection cannot diagnose. A blank confirms that the system is uncontaminated; however, it provides no information on whether the LC retention is reproducible, the mass calibration is holding, or the electrospray source is stable. Our results suggest that a curated 13-peptide standard fills this gap, as a single triplicate-injection set probed mass accuracy, retention time, and peak area across the full chromatographic and ionization range that a tryptic digest would occupy. Thus, the resulting pass-or-fail assessment is not an ancillary measurement but rather the prerequisite that allows MAM to move from a sensitive research method to a routinely defensible quality control tool [4,10,20,21]. This has several implications for laboratories implementing MAM, as the readiness of the platform can be asserted before, rather than inferred after sample analysis.

### 3.2. Alignment with USP 1060 and Regulatory Expectations

Designing the 13-peptide standard against USP General Chapter 1060 was a deliberate choice. USP 1060 sets the expectations for LC-MS system suitability across TIC intensity, mass accuracy, MS resolution, retention time, chromatographic resolution, and integrated peptide area, among other dimensions. By mapping each peptide in the mixture to one or more of these suitability parameters, the acceptance criteria are given a compendial foundation rather than an ad hoc one [22]. Furthermore, the OpenLab CDS environment in which the workflow runs already provides audit trail, electronic signature, and method version control consistent with GMP and FDA expectations for data integrity in computerized systems [23,24]. The automated result report appears to streamline documentation while reducing transcription error, and these features together position the workflow for adoption in regulated MAM environments without requiring bespoke documentation development by the user.

### 3.3. Operational Implications for Routine MAM

A typical implementation of the workflow would involve a daily system-suitability assessment before each MAM sample set. Three triplicate injections at roughly 27 min each, plus equilibration, return a verdict in under an hour of analytical time, and the result report is generated within OpenLab CDS without manual data review. Two operational consequences appear most relevant. First, the analyst obtains evidence that the LC, MS, and data processing pipeline are within specification before sample injection, which most likely reduces the risk of investing time in samples that would later be invalidated by the retrospective detection of system issues. Second, the verdict and the per-peptide metrics are preserved in OpenLab CDS as a per-session record that can be revisited as part of a root cause analysis when a downstream result is questioned. Thus, the workflow shifts MAM operations from a reactive posture, in which system performance is judged after the fact, to a proactive one, in which performance is established before sample analysis.

### 3.4. Future Directions

Several extensions of the workflow are in progress. Longitudinal SPC trending will aggregate the per-session metrics across days, operators, and instruments into control charts, which may flag column aging, source contamination, and calibration drift before they reach a product sample and thereby turn the daily verdict into a continuous record of platform performance and health. Application to therapeutic protein samples is the natural next step, beginning with monoclonal antibody tryptic digests and extending to more complex modalities such as antibody–drug conjugates, fusion proteins, and adeno-associated virus capsid proteins [2,3,25,26]. A key objective of these studies will be to test whether an SPC pass on a given day is predictive of MAM result quality for the samples analyzed thereafter. Further studies are needed to establish this predictive relationship and to integrate SPC outputs with laboratory information management systems, which would allow the verdict to directly gate downstream sample analysis. These extensions rest on the same analytical foundation described here. A passing SPC measurement indicates that the platform is in a state suitable for MAM analysis on the day it is acquired; aggregating these per-session measurements over time will additionally support trending of platform performance, which may aid in scheduling preventative maintenance before drift reaches a product sample.

### 3.5. Limitations

This work is a single-session feasibility demonstration, and its scope should be read accordingly. The data were acquired in one analytical session on a recently installed 6230C LC/TOF fitted with a new peptide-mapping column; the supplier notes that the standard may require a short column break-in before it performs as expected on a new system, so day-to-day, operator-to-operator, column-to-column, and instrument-to-instrument reproducibility remain to be established and are the subject of ongoing work. Carry-over was not formally assessed in this evaluation. Peak-area %RSD was configured for three representative targets rather than all thirteen; because absolute signal declined modestly across the injection sequence, extending an area-reproducibility criterion to every target, and across multiple sessions, is a needed refinement, and the per-replicate areas in Table S1 allow this to be evaluated for the remaining targets. We do not claim that the remaining targets would all meet a 10% peak-area %RSD criterion; their individual per-replicate areas are provided in Table S1 so that this can be inspected directly. The workflow has not yet been applied to a therapeutic protein digest; extension to monoclonal antibody and other biotherapeutic samples is the natural next step and is required before an SPC pass can be linked quantitatively to downstream MAM result quality.

## 4. Materials and Methods

### 4.1. Reagents and Standards

LC-MS grade water (Agilent InfinityLab Water for LC/MS, 1 L; P/N 5191-5121-001) and acetonitrile (Agilent InfinityLab Acetonitrile for LC/MS, 1 L; P/N 5191-5101-001) were from Agilent Technologies (Santa Clara, CA, USA). Formic acid (≥99% purity, Optima L The 13-peptide LC/MS reference standard (Agilent 13-Peptide LC/MS Standard; P/N 5191-4572) was supplied by Agilent Technologies (Santa Clara, CA, USA). The mixture spans the LC-MS system-readiness dimensions defined in USP General Chapter <1060> (Section 6.1, Table 6, “Common System Readiness Metrics”) [22], namely TIC signal intensity, mass accuracy, MS resolution, retention time, chromatographic resolution, and integrated peak area, and adds two features specific to biotherapeutic MAM workflows: an isotopically labeled peptide ALPAPIEK* (^13^C_6_, ^15^N_2_ lysine; monoisotopic mass 845.5104 Da) spiked at 1:100 with the light counterpart to provide a single-point, low-abundance response check and a deamidated peptide pair (VVSVLTVLHQDWLNGK and VVSVLTVLHQDWLDGK) used to monitor a deamidation event. Peptide sequences and individual diagnostic purposes are listed in Table 2. Two of the readiness dimensions in USP <1060>, in-source fragmentation and chromatographic resolution, are assessed through peptide selection rather than a separate numeric criterion: ALELFR, a fragile peptide prone to in-source fragmentation, serves as a source-conditions diagnostic, and the closely eluting native and deamidated forms of the VVSVLTVLHQDWLNGK and VVSVLTVLHQDWLDGK pair report on chromatographic separation. Their contribution to the pass-or-fail outcome is captured through the mass-accuracy, retention-time, and relative-area criteria applied to those peptides. The heavy-to-light spike provides a single-point, low-abundance response check rather than a characterization of detector dynamic range: reliable detection of the heavy peptide at 1:100 of the light peptide confirms that the system still responds to a low-abundance analyte, whereas loss of the heavy peptide at this ratio would flag a decline in low-level response and prompt the instrument to be stopped for detector recalibration or service. The heavy and light peptides are synthesized and purified individually and are combined at the specified ratio when the peptide mix is formulated; the acceptance range applied to the observed relative ratio (Section 4.4.2) is set to accommodate lot-to-lot variation in that formulation. The full SPC workflow comprises standard reconstitution (Section 4.2) followed by LC-MS acquisition under conditions optimized for this 13-peptide mixture (Section 4.3). The acquisition parameters were tuned to deliver a stable signal across all 13 peptides so that source health, mass accuracy, retention, and peak-area reproducibility could be verified simultaneously in a single-injection set. Sodium and iron adducts of selected peptides are monitored within the same method as additional-parameter peptides to demonstrate the workflow’s sensitivity to metal contamination.

### 4.2. Sample Preparation

The lyophilized peptide reference standard was reconstituted in 200 µL of 5% acetonitrile and 1% formic acid in LC-MS grade water to a working concentration of 1 pmol/µL per peptide, vortexed, and inverted to ensure complete dissolution. The reconstituted standard was stored at 4 °C between sessions, and injections were drawn directly from the reconstituted stock without further dilution. To check lot-to-lot consistency of the reference standard, a one-month-old lot was compared against a newly received lot, and all 13 peptides were detected in both.

### 4.3. Liquid Chromatography–Mass Spectrometry

#### 4.3.1. Chromatographic Conditions

Separations were performed on an Agilent 1290 Infinity III Bio LC system (Agilent Technologies, Santa Clara, CA, USA) with an Agilent AdvanceBio Peptide Mapping C18 column (2.1 × 150 mm, 2.7 µm; P/N 653750-902). Mobile phase A was 0.1% formic acid in LC-MS grade water; mobile phase B was 0.1% formic acid in LC-MS grade acetonitrile. Column temperature was held at 50 °C and flow rate was 0.4 mL/min. The gradient was: 0.00 to 2.00 min, 2% B; 2.00 to 20.00 min, 2 to 35% B; 20.00 to 21.00 min, 35 to 80% B; 21.00 to 22.00 min, 80% B; 22.00 to 23.00 min, 80 to 2% B; 23.00 to 27.00 min, 2% B. Injection volume was 1 µL, delivering 1 pmol of each peptide on column.

#### 4.3.2. Mass Spectrometry Conditions

Mass spectrometric detection was on an Agilent 6230C LC/TOF instrument (Agilent Technologies, Santa Clara, CA, USA) with a Dual Agilent Jet Stream electrospray ionization source operating in positive ion mode. The instrument was tuned and calibrated prior to acquiring the SPC injection set following the manufacturer’s standard procedure; the SPC workflow is intended as a readiness assessment that complements, and does not replace, instrument tune and calibration. The 6230C LC/TOF had been recently installed at the time of this evaluation; its manufacturer sensitivity specification is a signal-to-noise ratio of 1000:1 for 1 pg of reserpine. Source and ion-optics parameters were: drying gas temperature 250 °C; drying gas flow 13 L/min; nebulizer 35 psi; sheath gas temperature 300 °C; sheath gas flow 12 L/min; capillary voltage 3000 V; nozzle voltage 0 V; fragmentor 45 V; skimmer 35 V; and octopole 1 RF amplitude 750 V. Data were acquired in MS-only mode over *m*/*z* 200 to 2200 at 3 spectra/s, with continuous reference mass correction enabled using *m*/*z* 121 and 922 to maintain mass accuracy across the run.

### 4.4. System Performance Check Workflow

#### 4.4.1. Workflow Architecture

The SPC workflow runs inside OpenLab CDS 3.0 (Agilent Technologies, Santa Clara, CA, USA) using the integrated MAM for OpenLab CDS. It consists of three pages. The result set page collects injection-level metadata (method, column lot, run time, injection number). The system performance page evaluates each peptide in the 13-peptide standard against the pre-defined acceptance criteria. The result report aggregates the per-peptide outcomes into a single pass-or-fail verdict that can be exported with the audit trail and electronic signature controls already present in OpenLab CDS [23,24]. Acceptance criteria, target masses, charge states, and integration parameters are stored as method-level settings and can be locked to a specific method version.

#### 4.4.2. Acceptance Criteria and Performance Metrics

The system performance method evaluates each target against a configurable set of acceptance criteria, so that the metrics applied to a given target can be matched to the diagnostic role it plays rather than applied uniformly across the panel. Three complementary metrics were used: mass accuracy of the monoisotopic precursor, retention-time relative standard deviation (%RSD) across the three replicate injections, and integrated peak-area %RSD across the three replicates. Mass accuracy and retention-time %RSD were applied to all twelve primary peptide targets, and peak-area %RSD was additionally applied to a subset of them (ALELFR, ALPAPIEK, and DTLMISR) as a check on quantitative signal reproducibility. Mass accuracy was evaluated against a ±10 ppm window for every target except the short, early-eluting peptide EYK, for which a ±13 ppm window was used, and ALELFR, for which the configured window was ±10.05 ppm; retention-time %RSD was evaluated against a ≤0.5% limit and peak-area %RSD against a ≤10% limit. The isotopically labeled heavy peptide (ALPAPIEK*) and the oxidized variant (DTLMISR + O) were confirmed by exact monoisotopic mass, and the sodium- and iron-adduct targets, the methionine-oxidation variant, and the asparagine deamidation pair were evaluated as additional parameters against relative-area ratio criteria (adduct-to-parent peak area <2%; methionine oxidation < 2%; heavy-to-light peak area 0.5 to 2.5%; and asparagine deamidation < 8%). The complete set of criteria applied to each target is reported in Table S1 and summarized in Figure 5. A target passed when every criterion applied to it was met, and the system passed only when all targets met all of their applied criteria. These limits were pre-specified as method-level settings before acquisition. USP <1060> prescribes the performance dimensions (Section 6.1, Table 6) but defines their criteria qualitatively (“within a specified range”) and directs users to establish the numeric limits (Section 6.3) [22]; the values applied here were set from the instrument’s specified mass-accuracy performance under continuous reference-mass correction (±10 ppm), the retention reproducibility of the Agilent 1290 Infinity III Bio platform (retention-time %RSD ≤ 0.5%, consistent with and more stringent than general chromatographic system-suitability conventions [27]), and the signal reproducibility required for MAM relative quantitation (peak-area %RSD ≤ 10%), consistent with established MAM and peptide-mapping practice. Mass accuracy reports on calibration stability, retention-time %RSD on chromatographic reproducibility and column performance, and peak-area %RSD on signal stability and the quantitative robustness that MAM requires to make confident CQA calls downstream.

### 4.5. Data Processing and Analysis

The triplicate-injection acceptance criteria (mass accuracy, retention-time %RSD, and peak-area %RSD) were evaluated within the MAM for OpenLab CDS in OpenLab CDS, which produced the per-peptide and aggregated pass-or-fail outputs reported in Section 2. In parallel, and outside the SPC evaluation itself, Agilent MassHunter Qualitative Analysis 12.0 (Agilent Technologies, Santa Clara, CA, USA) with Find by Formula (FbF) was used to confirm peptide identifications against retention time, accurate mass, isotope pattern, and chromatographic peak shape. For the deamidation pair, extracted ion chromatograms were generated for both VVSVLTVLHQDWLNGK and VVSVLTVLHQDWLDGK at the [M+2H]^2+^ and [M+3H]^3+^ charge states to monitor the retention and signal of each peptide across the replicate injections rather than relying on the total ion chromatogram, which would have masked any co-elution.

## 5. Conclusions

The objective of this work was to evaluate a system performance check workflow as a readiness assessment for MAM analysis on an Agilent 6230C LC/TOF, using a 13-peptide reference standard designed against USP 1060. Across triplicate injections, all thirteen peptides in the standard met every acceptance criterion applied to them: mass accuracy and retention-time %RSD for the twelve primary targets, peak-area %RSD for a subset, and relative-area criteria for the adduct, oxidation, heavy/light, and deamidation monitors. Furthermore, the deamidation-pair peptides VVSVLTVLHQDWLNGK and VVSVLTVLHQDWLDGK were detected and reproducibly monitored at the +2 and +3 charge states across replicate injections (deamidation level 6.7 to 7.8%, criterion < 8%), demonstrating reproducible relative monitoring of a clinically meaningful post-translational modification. The result report generated by the MAM for OpenLab CDS returned a single pass verdict for the injection set, supporting documentation of system readiness, including the audit trail and electronic signatures, ahead of sample analysis. Our results suggest that the platform, the standard, and the workflow described here provide a practical foundation for routine MAM operations, and further studies applying the workflow to therapeutic protein samples and to longitudinal trending across instruments will establish its value in a production setting.

## Figures and Tables

**Table 1:** Representative system-suitability performance for peptides spanning the principal diagnostic categories of the 13-peptide standard, evaluated across triplicate injections on the Agilent 6230C LC/TOF. Mean retention time and mean mass accuracy are averaged over the three replicate injections; %RSD values are reported by the MAM for OpenLab CDS. The complete per-peptide evaluation for all 13 peptides is shown in Figure 4. The heavy internal standard (ALPAPIEK*) and the oxidized variant (DTLMISR + O) are monitored by exact mass; for these two rows, the value shown is the measured monoisotopic (neutral) mass in daltons rather than a ppm deviation, and peak-area %RSD is not applied to the low-abundance heavy peptide. The FSGVPSR + Na and ALPAPIEK + Fe rows monitor sodium and iron adducts as additional-parameter peptides within the SPC method; for these rows, the value in the Mass Accuracy column is the theoretical monoisotopic mass of the adduct in daltons, and the acceptance criterion is the adduct-to-parent area ratio (<2%), which was met across all three replicates.

Peptide (Charge)	Diagnostic Role	Mean RT (min)	RT %RSD	Mass Accuracy	Area %RSD	Result
ALELFR [M+2H]^2+^	In-source fragmentation	13.08	0.04%	8.99 ppm	8.09%	Pass
DTLMISR [M+2H]^2+^	Methionine oxidation	9.95	0.04%	8.59 ppm	8.04%	Pass
DTLMISR+O [M+2H]^2+^	Oxidized variant	8.74	—	850.428 Da	—	Pass
ALPAPIEK [M+2H]^2+^	Low-abundance response (light)	9.85	0.02%	8.60 ppm	8.40%	Pass
ALPAPIEK* [M+2H]^2+^	Low-abundance response (heavy, 1:100)	9.85	0.16%	845.517 Da	—	Pass
FSGVPSR + Na [M+2H]^2+^	Sodium adduct (contamination probe)	7.80	—	770.379 Da	—	Pass
ALPAPIEK + Fe [M+2H]^2+^	Iron adduct (contamination probe)	9.82	—	892.424 Da	—	Pass

ALPAPIEK* denotes the isotopically labeled heavy internal standard (^13^C_6_, ^15^N_2_ lysine).

**Table 2:** Composition and diagnostic purpose of the 13-peptide LC/MS reference standard.

#	Sequence	Diagnostic Purpose
1	VSK	Short, hydrophilic; elutes near the void volume. Front-end gradient marker.
2	EYK	Short, hydrophilic; conserved in IgG1, relevant to mAb workflows.
3	SNFR	Short, hydrophilic; extends low-molecular-weight coverage at gradient start.
4	ALELFR	Fragile myoglobin tryptic peptide, prone to in-source fragmentation. Source-conditions diagnostic.
5	FSGVPSR	Non-conserved IgG peptide; mid-gradient elution, reports mid-region chromatography.
6	DTLMISR	Methionine-containing, oxidation-prone. Monitors column aging and instrument drift.
7	ALPAPIEK	Forms iron adducts; selectivity differs 40 vs. 60 °C. Light half of the detector dynamic-range pair.
8	ALPAPIEK*	Heavy lysine (^13^C_6_, ^15^N_2_; +8 Da), spiked 1:100 for a single-point, low-abundance response check.
9	VLQPGYLEVDY	Tryptic peptide from mouse IgSF6; forms iron adducts. Second metal-contamination probe.
10	VVSVLTVLHQDWLNGK	Unmodified IgG1 peptide; asparagine prone to deamidation.
11	VVSVLTVLHQDWLDGK	Deamidated counterpart of peptide 10; the pair probes a deamidation event.
12	TVAAPSVFIFPPSDEQLK	Proline-rich; poor peak shape below 60 °C. Sensitive column-oven diagnostic.
13	VDNALQSGNSQESVTEQDSK	Longer IgG peptide, mid-gradient. Reports behavior of larger tryptic peptides.

## Data Availability

The original contributions presented in this study are included in the article/Supplementary Materials. Further inquiries can be directed to the corresponding authors.

## Supplementary Materials

The following supporting information can be downloaded at: https://www.mdpi.com/article/10.3390/ph19081240/s1, Table S1: Per-replicate system-suitability results for the thirteen peptides (mass accuracy and retention time); Table S2: Additional-parameter relative-area ratios (adducts, oxidation, heavy internal standard, and deamidation pair); Table S3: Per-replicate response for the deamidation-pair peptides and the mass-tagged monitors; Figure S1: MAM for OpenLab CDS System Performance report—results page; Figure S2: MAM for OpenLab CDS System Performance report—result report page.

## Abbreviations

The following abbreviations are used in this manuscript:

AAV	Adeno-Associated Virus
ADC	Antibody-Drug Conjugate
CQA	Critical Quality Attribute
EIC	Extracted Ion Chromatogram
FbF	Find by Formula
GMP	Good Manufacturing Practice
LC-MS	Liquid Chromatography–Mass Spectrometry
mAb	Monoclonal Antibody
MAM	Multi-Attribute Method
NPD	New Peak Detection
PTM	Post-Translational Modification
QC	Quality Control
RSD	Relative Standard Deviation
SPC	System Performance Check
SST	System-Suitability Test
TIC	Total Ion Chromatogram
USP	United States Pharmacopeia
