# Supplementary material for: System Performance Check (SPC): A Readiness Companion to Multi-Attribute Method (MAM) Analysis in Biopharmaceutical Quality Control
Source: Pharmaceuticals (Basel). 2026 Aug 6;19(8):1240. doi: 10.3390/ph19081240 (PMC13517073; doi:10.3390/ph19081240)

Supplementary Materials

System Performance Check (SPC): A Readiness Companion to Multi-Attribute Method (MAM) Analysis in Biopharmaceutical Quality Control

Manuscript ID: pharmaceuticals-4441399

All values are the per-injection results reported by the System Performance Check workflow in OpenLab CDS for triplicate injections (rep001-rep003) of the 13-peptide standard acquired in a single analytical session on the Agilent 6230C LC/TOF. %RSD is reported for each metric to which an acceptance criterion was applied.

Table S1. Per-replicate system-suitability results for the thirteen peptides (mass accuracy and retention time)

Peptide (charge)	Injection	Area	Height	Mass accuracy	RT (min)	Result
ALELFR (+2)	rep001	4,616,655.71	752,440	7.67 ppm (±10.05)	13.09	Pass
ALELFR (+2)	rep002	4,438,071.06	735,132	9.28 ppm (±10.05)	13.07	Pass
ALELFR (+2)	rep003	3,807,719.77	625,498	10.02 ppm (±10.05)	13.08	Pass
ALELFR (+2)	%RSD	8.09% (≤10%)			0.04% (≤0.5%)	Pass
ALPAPIEK (+2)	rep001	4,205,151.39	797,512	7.58 ppm (±10)	9.86	Pass
ALPAPIEK (+2)	rep002	4,054,932.45	764,422	9.44 ppm (±10)	9.85	Pass
ALPAPIEK (+2)	rep003	3,447,254.09	654,474	8.79 ppm (±10)	9.85	Pass
ALPAPIEK (+2)	%RSD	8.40% (≤10%)			0.02% (≤0.5%)	Pass
DTLMISR (+1)	rep001	978,397.86	207,405	2.99 ppm (±10)	9.94	Pass

Peptide (charge)	Injection	Area	Height	Mass accuracy	RT (min)	Result
DTLMISR (+1)	rep002	844,976.04	185,191	4.83 ppm (±10)	9.95	Pass
DTLMISR (+1)	rep003	634,859.61	137,776	4.46 ppm (±10)	9.95	Pass
DTLMISR (+1)	%RSD				0.02% (≤0.5%)	Pass
DTLMISR (+2)	rep001	8,088,457.71	1,392,321	7.74 ppm (±10)	9.96	Pass
DTLMISR (+2)	rep002	7,786,929.82	1,336,392	8.98 ppm (±10)	9.95	Pass
DTLMISR (+2)	rep003	6,681,565.66	1,170,518	9.05 ppm (±10)	9.95	Pass
DTLMISR (+2)	%RSD	8.04% (≤10%)			0.04% (≤0.5%)	Pass
EYK (+1)	rep001	227,823.13	54,381	10.37 ppm (±13)	1.29	Pass
EYK (+1)	rep002	203,823.08	50,677	11.32 ppm (±13)	1.29	Pass
EYK (+1)	rep003	159,774.69	38,368	11.48 ppm (±13)	1.28	Pass
EYK (+1)	%RSD				0.36% (≤0.5%)	Pass
FSGVPSR (+2)	rep001	2,549,094.47	511,482	8.40 ppm (±10)	7.83	Pass
FSGVPSR (+2)	rep002	2,477,197.12	491,467	7.43 ppm (±10)	7.83	Pass
FSGVPSR (+2)	rep003	2,102,934.63	413,530	9.38 ppm (±10)	7.83	Pass
FSGVPSR (+2)	%RSD				0.01% (≤0.5%)	Pass
SNFR (+2)	rep001	1,609,610.93	191,347	8.54 ppm (±10)	3.56	Pass
SNFR (+2)	rep002	1,597,481.03	176,286	9.53 ppm (±10)	3.54	Pass
SNFR (+2)	rep003	1,370,813.03	153,052	7.72 ppm (±10)	3.53	Pass
SNFR (+2)	%RSD				0.33% (≤0.5%)	Pass

Peptide (charge)	Injection	Area	Height	Mass accuracy	RT (min)	Result
TVAAPSVFIFPPSDEQLK (+3)	rep001	1,582,130.73	87,203	6.82 ppm (±10)	16.84	Pass
TVAAPSVFIFPPSDEQLK (+3)	rep002	1,446,587.23	79,135	9.04 ppm (±10)	16.84	Pass
TVAAPSVFIFPPSDEQLK (+3)	rep003	1,211,085.80	67,850	7.86 ppm (±10)	16.83	Pass
TVAAPSVFIFPPSDEQLK (+3)	%RSD				0.01% (≤0.5%)	Pass
VDNALQSGNSQESVTEQ DSK (+3)	rep001	757,166.13	157,179	5.91 ppm (±10)	8.07	Pass
VDNALQSGNSQESVTEQ DSK (+3)	rep002	763,414.98	156,382	6.25 ppm (±10)	8.07	Pass
VDNALQSGNSQESVTEQ DSK (+3)	rep003	616,054.82	122,011	5.98 ppm (±10)	8.07	Pass
VDNALQSGNSQESVTEQ DSK (+3)	%RSD				0.01% (≤0.5%)	Pass
VLQPGYLEVDY (+2)	rep001	1,877,575.50	307,544	5.64 ppm (±10)	14.99	Pass
VLQPGYLEVDY (+2)	rep002	1,769,410.38	291,986	6.71 ppm (±10)	14.98	Pass
VLQPGYLEVDY (+2)	rep003	1,566,716.53	260,686	6.51 ppm (±10)	14.99	Pass
VLQPGYLEVDY (+2)	%RSD				0.01% (≤0.5%)	Pass
VSK (+1)	rep001	399,303.47	154,901	3.77 ppm (±10)	0.89	Pass
VSK (+1)	rep002	348,242.02	138,913	5.55 ppm (±10)	0.90	Pass
VSK (+1)	rep003	260,006.33	102,001	4.25 ppm (±10)	0.89	Pass

Peptide (charge)	Injection	Area	Height	Mass accuracy	RT (min)	Result
VSK (+1)	%RSD				0.19% (≤0.5%)	Pass
VVSVLTVLHQDWLNGK (+3)	rep001	12,406,953.73	1,937,097	8.58 ppm (±10)	17.47	Pass
VVSVLTVLHQDWLNGK (+3)	rep002	11,458,687.32	1,845,307	9.56 ppm (±10)	17.47	Pass
VVSVLTVLHQDWLNGK (+3)	rep003	10,409,084.93	1,714,537	9.61 ppm (±10)	17.47	Pass
VVSVLTVLHQDWLNGK (+3)	%RSD				0.01% (≤0.5%)	Pass

The deamidation-pair peptide VVSVLTVLHQDWLDGK is the thirteenth peptide; it is monitored at the +2 and +3 charge states and quantified as a relative-area ratio (Table S2), with per-injection response reported in Table S3.

Table S2. Additional-parameter relative-area ratios (adducts, oxidation, heavy internal standard, and deamidation pair)

Target	Group	rep001	rep002	rep003	Criterion	Result
ALPAPIEK (+2)	Iron adduct	0.00%	0.00%	0.00%	<2%	Pass
ALPAPIEK* (+2)	Heavy/light ratio	0.82%	0.81%	0.80%	0.5–2.5%	Pass
DTLMISR+O (+2)	Methionine oxidation	1.37%	1.26%	1.24%	<2%	Pass
FSGVPSR (+2)	Sodium adduct	0.00%	0.00%	0.00%	<2%	Pass
VVSVLTVLHQDWLDGK (+2)	Deamidation pair	6.75%	7.04%	6.70%	<8%	Pass
VVSVLTVLHQDWLDGK (+3)	Deamidation pair	7.81%	7.75%	7.49%	<8%	Pass

The heavy/light ratio provides a low-abundance relative-response check; the deamidation-pair relative area is a reproducible relative measurement of the deamidation level (deamidated / [deamidated + native]).

Table S3. Per-replicate response for the deamidation-pair peptides and the mass-tagged monitors

Peptide (charge)	Injection	Area	Height	Measured mass	RT (min)	Result
VVSVLTVLHQDWLDGK (+2)	rep001	151,048.05	26,706	1807.99	17.75	Pass
VVSVLTVLHQDWLDGK (+2)	rep002	137,564.21	23,719	1807.99	17.75	Pass
VVSVLTVLHQDWLDGK (+2)	rep003	107,951.55	19,143	1807.99	17.75	Pass
VVSVLTVLHQDWLDGK (+3)	rep001	1,051,672.09	177,575	1808.00	17.75	Pass
VVSVLTVLHQDWLDGK (+3)	rep002	962,497.69	157,767	1808.00	17.75	Pass
VVSVLTVLHQDWLDGK (+3)	rep003	842,226.65	143,078	1808.00	17.75	Pass
VVSVLTVLHQDWLNGK (+2)	rep001	2,085,539.78	350,727	1807.01	17.47	Pass
VVSVLTVLHQDWLNGK (+2)	rep002	1,816,199.06	310,681	1807.01	17.48	Pass
VVSVLTVLHQDWLNGK (+2)	rep003	1,502,414.39	254,325	1807.01	17.47	Pass
ALPAPIEK* (+2) heavy	rep001	34,687.04	6,389	845.52	9.87	Pass
ALPAPIEK* (+2) heavy	rep002	32,951.38	6,129	845.52	9.84	Pass
ALPAPIEK* (+2) heavy	rep003	27,851.40	5,441	845.52	9.85	Pass

Peptide (charge)	Injection	Area	Height	Measured mass	RT (min)	Result
DTLMISR+O (+2) oxidized	rep001	112,236.81	21,022	850.43	8.73	Pass
DTLMISR+O (+2) oxidized	rep002	99,053.60	18,264	850.43	8.74	Pass
DTLMISR+O (+2) oxidized	rep003	83,575.73	16,511	850.43	8.74	Pass

Acceptance for the deamidation-pair and mass-tagged monitors is evaluated by relative-area ratio (Table S2) rather than by parts-per-million mass accuracy; the measured mass column confirms target identity.

Figure S1. MAM for OpenLab CDS System Performance report — results page

The software-generated System Performance report page from the MAM for OpenLab CDS System Performance Check workflow, showing the per-target pass/fail summary across the triplicate injections. These screenshots are provided here as Supplementary Materials; the quantitative results they summarize are presented in the main-text figures and in Tables S1–S3.

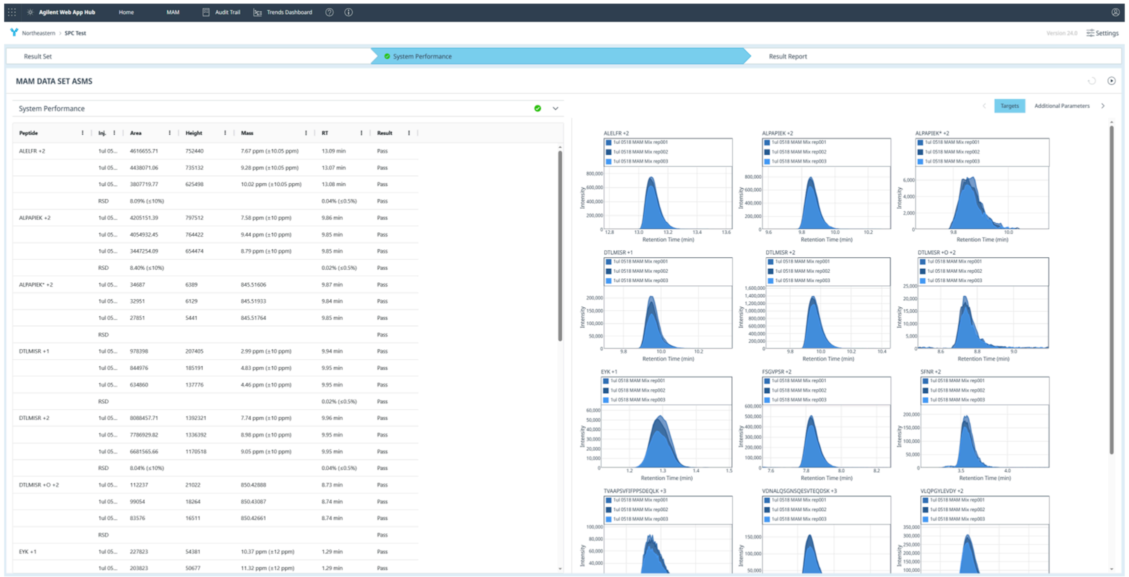

Figure S2. MAM for OpenLab CDS System Performance report — result report page

The software-generated Result Report page from the same workflow, showing the aggregated pass/fail verdict and the associated audit-trail and electronic-signature metadata captured in OpenLab CDS.

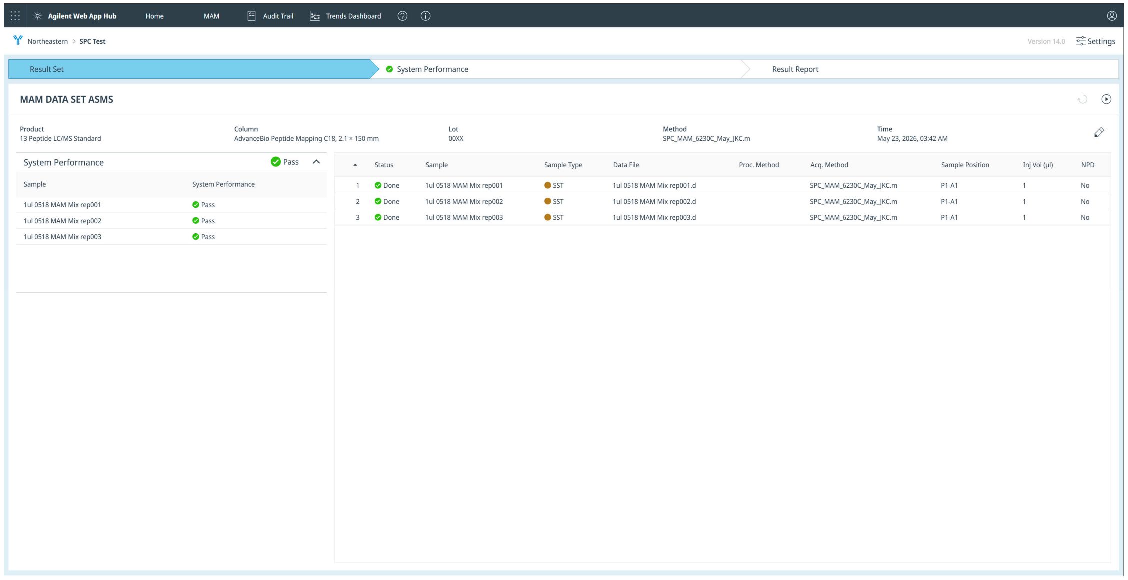

Supplement: Supplementary file 1 [file pharmaceuticals-19-01240-s001.zip › pharmaceuticals-4441399-supplementary.pdf]
